# Medial Rectus Disinsertion for the Management of Large-Angle Sensory Esotropia

**DOI:** 10.3390/medicina60071104

**Published:** 2024-07-06

**Authors:** So Young Han, Bo Young Chun, Hye Jin Lee, Hyun Kyung Kim, Mi Sun Kwon, Ho Seok Lee, Soolienah Rhiu

**Affiliations:** 1 Department of Ophthalmology, Kangbuk Samsung Hospital, Sungkyunkwan University School of Medicine, Seoul 03181, Republic of Korea; 2 Department of Ophthalmology, School of Medicine, Kyungpook National University, Daegu 41944, Republic of Korea; byjun424@hotmail.com (B.Y.C.);; 3 Brain Science & Engineering Institute, School of Medicine, Kyungpook National University, Daegu 41944, Republic of Korea; 4 Department of Ophthalmology, Chuncheon Sacred Heart Hospital, College of Medicine, Hallym University, Chuncheon 24253, Republic of Korea; 5 Department of Ophthalmology, Jeju National University Hospital, Jeju National University School of Medicine, Jeju 63241, Republic of Korea; 6 Department of Ophthalmology, Hangil Eye Hospital, Catholic Kwandong University College of Medicine, Incheon 21388, Republic of Korea; 7 Department of Ophthalmology, Dongtan Sacred Heart Hospital, College of Medicine, Hallym University, Hwaseong-si 18450, Republic of Korea

**Keywords:** esotropia, medial rectus disinsertion, strabismus

## Abstract

*Background and Objectives*: The aim of the report is to report the outcomes of the medial rectus (MR) disinsertion procedure for the management of large-angle esotropia (ET) patients. *Materials and Methods*: This is a retrospective case series of patients with large-angle ET who underwent an MR disinsertion procedure between March 2012 to April 2022. The procedure happened accidentally during muscle surgery. The demographic and clinical data, including sex, age, visual acuity, pre- and postoperative angle of strabismus, duction limitations, results of intraoperative forced duction tests, and follow-up duration were collected from medical records. *Results*: Five patients were enrolled in this study. The mean age was 62.2 ± 9.8 years, and the mean follow-up was 24.8 ± 8.7 months. The ET at the primary position of gaze was 92.0 ± 17.9 prism diopters (PD) before MR disinsertion and 38.0 ± 29.5 PD after MR disinsertion only. Abduction deficiency was −4 before after MR disinsertion, which improved to −1 at the last follow-up. *Conclusions*: The results of MR disinsertion were not as frustrating as anticipated. MR disinsertion may be considered in patients with large-angle sensory ET who refuse surgery on the opposite eye.

## 1. Introduction

The management of large-angle esotropia (ET) is challenging. There is no consensus on how many muscles should be operated on for the correction of large-angle ET. In esodeviation cases with a deviation angle of more than 60 prism diopters (PD), most ophthalmologists consider performing three-muscle surgery—bilateral medial rectus (MR) muscle recession combined with resection of the non-dominant lateral rectus (LR) muscle [1,2]. However, the operation time is longer for three-muscle surgery and postoperative limitations of ductions may occur [1,2]. In addition, patients with sensory ET often refuse to have their dominant eyes operated on. This study describes the surgical outcomes of unintentional MR disinsertion procedures for the correction of large-angle ET.

## 2. Materials and Methods

The study was approved by the local IRB committee and followed the tenets of the Declaration of Helsinki. Informed consent was obtained from all of the patients before surgery.

This retrospective case series included patients with ET of >60 PD who had undergone MR disinsertion from March 2012 to April 2022. Patients with thyroid eye disease, the existence of an A-V pattern, and a history of prior strabismus or botulinum toxin injection were excluded. The deviation angle was measured in the primary position of gaze using the modified Krimsky method at 33 cm.

The preoperative data collected included age, gender, onset of strabismus (childhood or adulthood), type of strabismus (including paralytic and restrictive), prior strabismus surgery on other muscles, and the limitation in ocular rotations in the field of gaze opposite the affected muscle. The recorded surgical data included forced duction testing (FDT) at the time of surgery (graded as no restriction, mild restriction, or moderate to severe restriction), and type of surgery. During surgery, the surgeon performed either a limbal or fornix incision, based on personal preference. In all cases, the tendon was exposed on a Jameson muscle hook.

## 3. Results

The mean age of patients was 62.2 ± 9.8 years and the mean follow up was 24.8 ± 8.7 months. The ET at the primary position of gaze was 92.0 ± 17.9 prism diopters (PD) before MR disinsertion occurred and the postoperative angle was 38.0 ± 29.5 PD after only MR disinsertion. Abduction deficiency was −4 before after MR disinsertion, which improved to −1 at the last follow-up. The characteristics of the patients are summarized in Table 1. 

Case 1 was a 46-year-old female presented with 100 PD ET in the right eye. Her visual acuity was hand motion in the right eye (spherical equivalent (SE) of the refractive error +5.0 D) and 20/20 in the left eye (SE, +1.0 D). She had been having ET since her childhood, but she never visited a hospital before. Severe abduction deficit (−4) of the right eye was observed. During strabismus surgery, severe restriction of the right MR by the forced duction test was noted. However, there was a sudden accidental MR disinsertion immediately after placing two Jameson muscle hooks in order to measure and mark the amount of MR recession prior to placing the suture in the MR tendon. After 6 h, her esodeviation decreased from 100 PD to 30 PD by MR disinsertion and there were no restrictions in the right MR. On the same day, the Hummelsheim operation was performed on the right eye. Two years after surgery, she was found to be orthophoric in the primary position of gaze with the limitation in adduction (−1.5).

**Figure 1:**
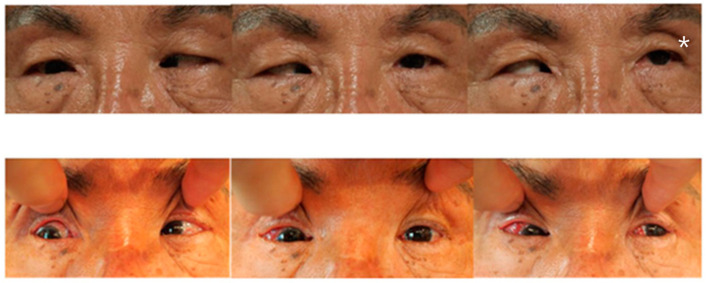
Photos of Case 1 preoperative (**upper column**) and 1 month postoperative (**lower**). A 63-year-old patient presented with 100 PD esotropia in the right eye. One month after medial rectus disinsertion and lateral rectus with a 7.0 mm resection of the right eye, he had esodeviation of 10 PD in the primary position of gaze with nearly normal adduction and abduction. * Abduction in left eye was complete preoperatively.

Case 2 was a 63-year-old male who presented with 100 PD ET in the right eye (Figure 1). His visual acuity was 20/200 in the right eye (SE, −0.5 D) and 20/20 in the left eye (SE, +0.5 D). Severe abduction deficit (−4) of the right eye was observed. Ten years before, the patient experienced a severe automobile accident. Optic atrophy of the right eye due to the traumatic optic neuropathy was observed during the fundus examination. Severe abduction deficit (−4) of the right eye was observed due to the sixth cranial nerve palsy that resulted from previous head trauma during the automobile accident. During strabismus surgery, severe restriction of the right MR by the forced duction test was noted, and the right MR was unintentionally disinserted during the procedure. After MR disinsertion, his esodeviation decreased from 100 PD to 30 PD and there was no restriction of the right MR. After two months, an additional right LR with a 7.0 mm resection was performed in the right eye. Two years after surgery, his esodeviation was 10 PD in the primary position of gaze without limitation in abduction or with little limited adduction (−1).

**Figure 2:**
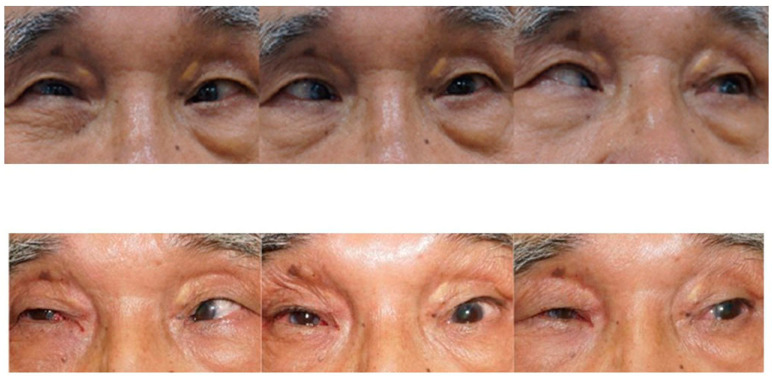
Photos of Case 3 preoperative (**upper column**) and 1 month postoperative (**lower**). A 72-year-old patient presented with 90 PD esotropia in the right eye. One month after Jenson operation and medial rectus disinsertion of the right eye, he was orthophoric in the primary position of gaze with nearly normal adduction and abduction.

Case 3 was a 72-year-old male presented with 90 PD ET in the right eye (Figure 2). His visual acuity was 20/400 in the right eye (SE, +0.5 D) and 20/20 in the left eye (SE, plano). Forty years ago, the patient experienced blunt trauma to the right eye during military service. Optic atrophy of the right eye due to the traumatic optic neuropathy was observed during the fundus examination. Severe abduction deficit (−4) of the right eye was observed due to sixth cranial nerve palsy. However, there was no restriction of the right MR by forced duction test during strabismus, and Jenson procedure of the right eye was completed. Three months after surgery, it was found that the Jensen procedure decreased his esodeviation from 90 PD ET to 60 PD ET. The patient refused strabismus surgery on the dominant eye to correct the residual ET. Then, MR of the right eye was disinserted accidentally, and this procedure decreased the esodeviation from 60 PD ET to orthophoric. Two years after MR disinsertion, he was orthophoric in the primary position of gaze without limitation in abduction or with little limited adduction (−1).

**Figure 3:**
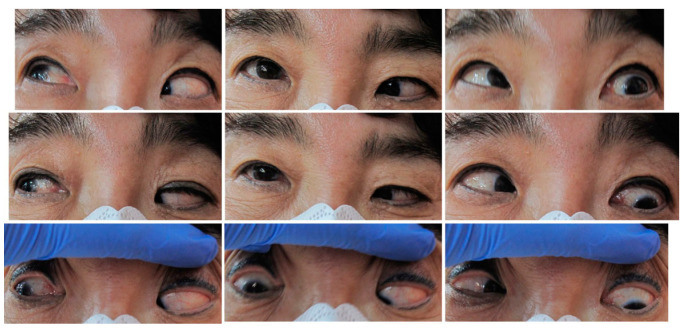
Photos of Case 4 preoperative esotropia in 9 cardinal directions. A 63-year-old patient presented with 100 PD esotropia in the left eye. Two weeks after medial rectus muscle disinsertion and lateral rectus muscle resection of the left eye, she was orthophoric and slightly hypotropic in the primary position of gaze with normal adduction and abduction.

**Figure 4:**
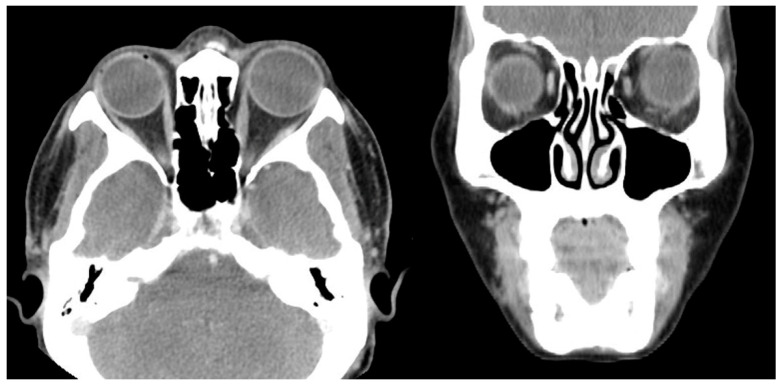
Postoperative CT image of Case 4. No definite abnormality is noted after left medial rectus muscle disinsertion.

Case 4 was a 63-year-old female who presented with 100 PD ET in the left eye (Figure 3). Her visual acuity was 20/20 in the right eye (SE, −0.25) and counting fingers in the left eye (SE, +2.25 D). She had been having ET since childhood, but has never visited a hospital before. Severe abduction deficit (−3) of the left eye was observed. During strabismus surgery, severe restriction of the left MR by the forced duction test was noted. However, there was a sudden accidental MR disinsertion immediately after placing two Jameson muscle hooks that were used to measure and mark the amount of MR recession prior to placing the suture in the MR tendon. One month after surgery, esodeviation was 50 PD with slight hypotropia in her left eye. Abduction deficit in the left eye was −2.0. Three months after surgery, orbit CT with enhancement was performed to check on the medial rectus status (Figure 4). Six months after primary surgery, left lateral rectus muscle with a 7.0 mm resection was performed. Two weeks after the secondary surgery, she was orthophoric and slightly hypotropic in the primary position of gaze with no limitation in adduction, which was maintained for two years.

**Figure 5:**
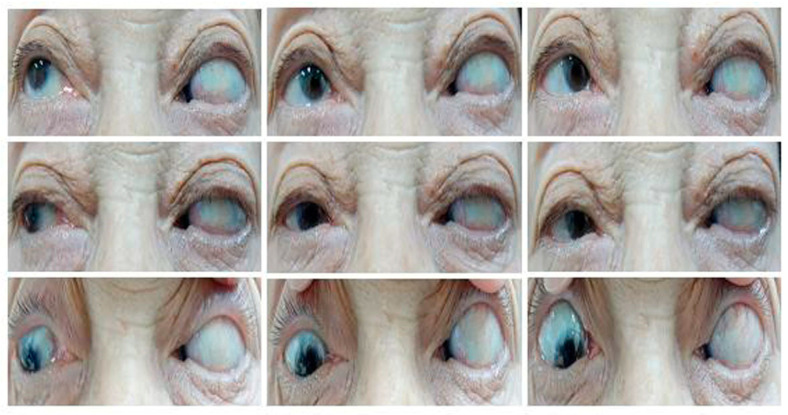
Photos of Case 5 preoperative esotropia in 9 cardinal directions. A 67-year-old patient presented with 100 PD esotropia in the left eye. One day after medial rectus muscle disinsertion she still had 100 PD esotropia in the left eye with severe abduction restriction.

**Figure 6:**
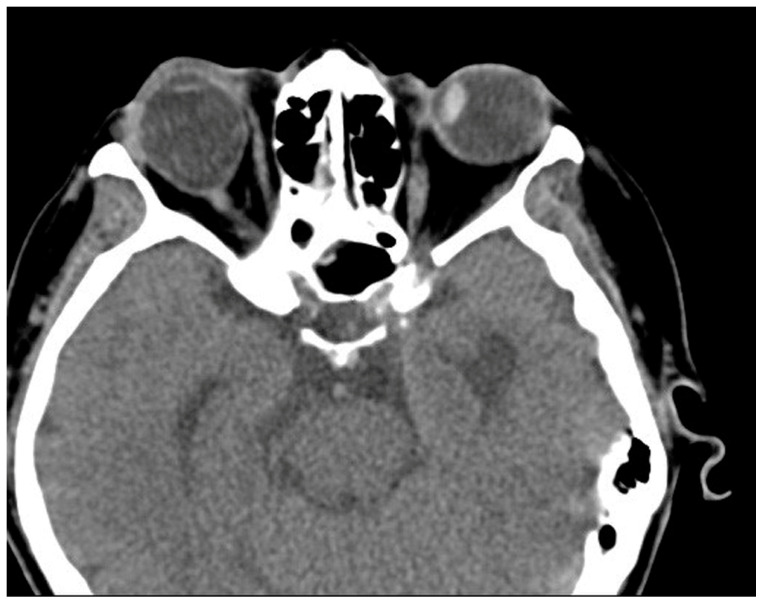
Preoperative CT image of Case 5. Thickening of the left medial rectus muscle with severe left esotropia is noted.

Case 5 was a 67-year-old female who presented with 100 PD ET in the left eye (Figure 5). Her visual acuity was 20/20 in the right eye (SE, −2.125) and hand motion in the left eye (SE, +1.0 D). Twenty years ago, the patient had blunt trauma to her head and lost vision in her left eye. Optic atrophy of the left eye due to traumatic optic neuropathy was observed during the fundus examination. Severe abduction deficit (−4) of the left eye was observed. Preoperative CT was performed and enlarged left medial rectus was noted (Figure 6). During strabismus surgery, severe restriction of the left MR by the forced duction test was noted and the left MR was disinserted accidentally during the procedure. After 1 day, her esodeviation was still 100 PD by MR disinsertion. One month after surgery, esodeviation was 80 PD in her left eye. The patient refused further surgery and was lost during follow-up.

## 4. Discussion

In this study, we report the surgical outcomes of an unintentional MR disinsertion procedure for the management of large-angle ET in five patients. The MR disinsertion procedure reduced esodeviation from 60 to 70 PD without causing severe limitation in adduction or abduction. During surgery, thin stiff muscles were noted and the muscles snapped while trying to isolate them with a muscle hook. 

Disinsertion of the extraocular muscle has been used by early strabismus surgeons and as well as more recently in certain conditions [3,4]. Bagheri et al. reported that MR disinsertion corrected esodeviation from 35 PD to 14.2 PD in patients with chronic complete sixth nerve palsy [4]. However, in our study, MR disinsertion corrected esodeviation 70 PD in 2 patients, 60 PD in 1 patient, 50 PD in 1 patient, and 20 PD in 1 patient. This difference in the amount of corrected ET by MR disinsertion is quite large, and it may be due to the function of the LR muscle. It is obvious that MR disinsertion will correct more esodeviation in the eye with normal abduction than in the eye with a limitation in abduction.

Our results correspond with a previous study that reported persistent eye movement following disinsertion of the extraocular muscle from its scleral insertion [5]. The authors speculated that the reason for the unexpected and persistent eye movement in the direction of the action of the disinserted muscle is due to the anatomic insertion involving the surrounding connective tissue, which allows the orbital portion to still exert a traction force on the eye [5]. They also reported that the power of connective tissue may explain, in part, the persistence of nearly normal adduction in the presence of an MR muscle slippage [5]. This is comparable to our results, which reported that the MR disinsertion procedure caused no or minimal limitation in adduction. It should also be considered that the MR might have reattached to the sclera. This makes predicting the outcome in terms of PD of the effect of MR disinsertion highly variable, depending on an individual patient’s anatomy. The location of reattachment might predict the amount of surgical correction that can be achieved. 

Our study had limitations due to its small number of patients. The average follow-up period was also only 2 years. Large MR recession using adjustable sutures would have been a more controlled approach for patients with large-angle ET with normal vision. However, all five patients in this study had very poor vision, which may have disabled the precise fixation of the operating eye during placement of the adjustable sutures. Another limitation of our study was that only a select number of cases had pre- and postoperative CT scans of the muscle. We did not plan to conduct imaging to assess the muscle condition, hence, MRI was not performed. For patients with a potential risk of disinsertion, it is recommended to obtain an MRI and a detailed eye examination prior to surgery. Finally, we were unable to evaluate the pre- and postoperative binocular function due to poor vision in the deviated eye. Therefore, we cannot apply our surgical outcomes in large-angle ET patients with normal vision. 

## 5. Conclusions

In conclusion, there was a marked improvement in esodeviation of 60 to 70 PD after MR disinsertion. Although unplanned MR disinsertion occurred accidentally during the surgery, it resulted in an unexpected and favorable outcome. In addition, there was minimal limitation in abduction or adduction after MR disinsertion. To the best of our knowledge, this is the first case series of MR disinsertion procedure in patients with large-angle ET with long-term follow up. This case series suggests that MR disinsertion, although unfortunate, can be an instructive situation. This highlights a course of action that can be reluctantly adopted in similar circumstances with large-angle sensory ET who refuse surgery on the dominant eye. However, considering that nearly all cases in this study involved sixth nerve palsy, it may not be appropriate to generalize the MR disinsertion results for large-angle sensory ET in patients with a healthy and robust lateral rectus muscle.

Even for highly experienced strabismus surgeons, there are instances where an extraocular muscle can be inadvertently disinserted during the procedure, which can be a catastrophic event for the surgeon in terms of anticipating the future clinical course and planning the subsequent management. In this article, we present five cases of accidental disinsertions of the MR muscle, with the aim of sharing valuable insights and contributing to the knowledge base on this rare but significant complication. In the event of accidental severance of the MR muscle during surgery, the findings from this study can provide guidance on the approximate clinical course and the degree of ET correction required. 

## Figures and Tables

**Table 1:** Characteristics of patients with large-angle sensory esotropia managed with medial rectus disinsertion.

Case	Sex/ Age	VA (OD)	VA (OS)	Preop. Angle (PD)	Postop. Angle after MR Disinsertion (PD)	Additional Surgery Performed	Postop. Angle at Last Follow-Up (PD)	Preop. Abduction Deficiency	Postop. Abduction Deficiency at Last Follow-Up	Postop. Adduction Deficiency
1	F/46	HM	20/20	100	30	Hummelsheim	0	−4	−1.5	−1.5
2	M/63	20/200	20/20	100	30	RLR Res 7.0 mm	10	−4	−1	−1
3	M/72	20/400	20/20	60	0	Jensen	0	−4	−1	−1
4	F/63	20/20	FC	100	50	LLR Res 7.0 mm	0	−3	0	0
5	F/67	20/20	HM	100	80	Follow-up loss	80	−4	−4	0

VA—visual acuity; PD—prism diopter; Preop—preoperative; postop—postoperative; MR—medial rectus muscle; RLR—right lateral rectus muscle; Res—resection.

## Data Availability

The datasets used and/or analyzed during the current study are available from the corresponding author upon reasonable request.
